# Groundwater in sedimentary basins as potential lithium resource: a global prospective study

**DOI:** 10.1038/s41598-021-99912-7

**Published:** 2021-10-26

**Authors:** Elza J. M. Dugamin, Antonin Richard, Michel Cathelineau, Marie-Christine Boiron, Frank Despinois, Anne Brisset

**Affiliations:** 1 GeoRessources, Université de Lorraine, CNRS, CREGU, Nancy, France; 2 TotalEnergies, Centre Scientifique et Technique Jean Féger, Pau, France

**Keywords:** Geochemistry, Hydrogeology

## Abstract

Electric cars will require to increase the production of lithium dramatically (up to 2 Mtons lithium equivalent carbonate per year by 2030). However, conventional hard-rock and salar mining are facing environmental and social concerns. Therefore, alternative lithium resources may help meeting the global demand for the next decades. Here, we provide a systematic analysis of published lithium concentration in about 3000 samples of groundwater from 48 sedimentary basins worldwide. The highest lithium concentrations (> 10^2^ mg l^−1^) are primarily found in high salinity waters (Total Dissolved Solids > 10^5^ mg l^−1^) and are in the same range as brines from the most productive salars. Conservative estimations based on fluid volume and lithium concentration in selected reservoirs indicate that these lithium resources are comparable to salars and hard-rock mines (0.1–10 Mtons lithium). Therefore, lithium in groundwater from sedimentary basins could be a significant potential resource for the next decades.

## Introduction

Lithium is widely used for energy storage (Li-ion rechargeable batteries, around 70% global lithium consumption) in ceramics, glass and lubricating grease^1^. The lithium demand had increased from 37 ktons of lithium in 2016 to 52 ktons of lithium in 2018. It is forecasted to exceed 0.38 Mtons of lithium by 2028 due to the increased need for rechargeable Li-ion batteries for electromobility^2^. Currently exploited primary lithium resources are found in hard rocks (granites, aplites and pegmatites) and closed basin brines (salars) (respectively about 26 and 58%)^3^. The total global resource is estimated to be 86 Mtons of lithium^1^. Although the current resources are far higher than the expected demand for the next ten years, momentary tensions may arise, linked to local difficulties in the mining operations. In particular, increasing environmental concerns and challenges in the social acceptance of salar exploitations (e.g. water use in desertic areas) may seriously affect future production^4^. In addition, energy costs and CO_2_ balance for mining and transportation of hard rocks may influence the sustainability of this type of operation^5^. Therefore, developing alternative lithium resources may significantly contribute to meeting the global demand.

Groundwaters in sedimentary basins have been widely recognized for carrying appreciable amounts of metals (hundreds to thousands of mg l^−1^) in their dissolved load^6^. The potential “ore-forming solutions”^7^ may also be considered “liquid ores”^8^. In the perspective of increasing lithium demand, groundwaters from high-enthalpy geothermal fields have recently attracted considerable attention for extracting lithium as a by-product of geothermal energy^9^ (e.g. Salton Trough^10^). Deep saline groundwaters in sedimentary basins could also be of interest but have not been studied in detail. Yet, sedimentary formation waters are usually produced in large volumes in oil and gas fields, low-enthalpy geothermal fields and CCUS (carbon capture, utilisation and storage) operations in saline reservoirs^11–13^. Lithium concentrations in these waters have been widely documented. However, a global analysis of the potential resource in lithium is still lacking. Here, a database of published lithium concentrations from sedimentary formation waters worldwide was built. The main features of the reservoirs (depth, temperature and age of host rocks) and chemical parameters, such as Total Dissolved Solids were examined to evaluate the first-order parameters controlling lithium concentrations in sedimentary formation waters as well as Mg/Li which is relevant for lithium recovery^14,15^. The lithium resource was calculated for selected lithium-rich reservoirs based on available lithium concentrations and conservative estimates of fluid volumes. The estimated lithium resources were compared to current lithium resources from hard rocks and salars to examine how they can face forecasted lithium demand.

### Data compilation and processing

**Figure 1:**
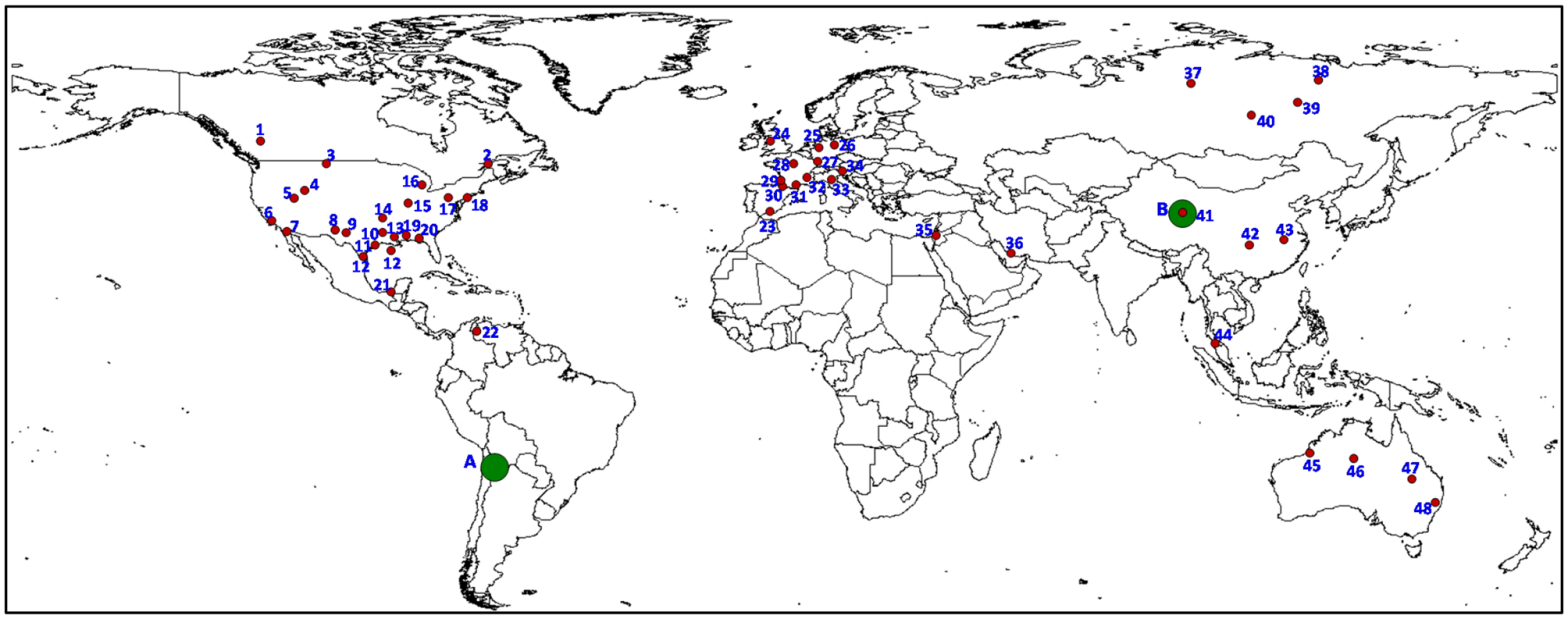
World map showing the sedimentary basins, salars and salt lakes with published data used in this study. Red dots: sedimentary formation waters; green circles: closed basin brines (salars and salt lakes). The numbers and capital letters in blue refer to the identification number in Table 1.

The database built for this work covers 48 sedimentary onshore and offshore basins worldwide, mainly from North America and Europe (Fig. 1, Table 1). Some of the selected basins are subjected to oil-gas-coal-shale-gas extraction, and exploitation for geothermal energy and CO_2_ storage in some others. Analyses of about 3000 individual samples of sedimentary formation waters were considered from published sources, the number of data ranging between 2 to 330 per basin (Table 1). All selected data correspond to reservoir waters (i.e. “formation waters”) and spring waters. According to international stratigraphic charts, the age of the reservoir host rocks spans from Proterozoic to Cenozoic^16^.

**Table 1:** Published sources used for building the database.

Countries	Basin name	Identification number	Number of samples	References
**Closed basin brines**				
Argentina	Puna—Atacama—Altiplano—Northwest	A	490	Ericksen et al. 1976; Ericksen and Salas 1987; Risacher and Fritz 1991; Risacher et al. 1999; Steinmetz et al. 2018; Gabriela et al. 2020
China	Qaidam	B	13	Qishun et al. 2010; Tan et al. 2011
**Sedimentary formation waters**				
Australia	Amadeus	46	26	Andrew et al. 2005
Australia	Canning	45	19	Ferguson et al. 2007
Australia	Eromanga	47	10	von Strandmann et al. 2014
Australia	Murray	48	42	Cartwright et al. 2004
Canada	Quebec	2	5	Pinti et al. 2011
Canada	West Canadian-Alberta	1	147	Connolly et al. 1990; Hitchon 1971; Eccles and Berhane 2011; Hitchon 2001
China	Jianghan	43	12	Yu et al. 2021
China	Qaidam	41	27	Qishun et al. 2010; Tan et al. 2011
China	Sichuan	42	92	Xun et al. 1997; Xun et al. 2018; Gao et al. 2020
England	Cheshire—Worcester	24	15	Tellam 1995
France	Aquitaine	29	81	Négrel et al. 2012
France	Gulf of Lion	31	6	Aquilina et al. 2002
France	Limagne	32	10	Millot and Négrel 2007
France	Paris	28	79	Michard and Bastide 1988; Fontes and Matray 1993; Millot and Négrel 2007 Linard et al. 2011; Millot et al. 2011; Castillo et al. 2015
France	Pyrenean Foothills	30	3	Millot and Négrel 2007
France-Germany	Rhine Graben	27	65	Pauwels et al. 1993; Aquilina et al. 1997; Millot and Négrel 2007; Sanjuan et al. 2010; Stober and Butcher 2015; Sanjuan et al. 2016; Sanjuan et al. 2020
Germany	Northwest German	26	45	Kloppmann et al. 2001; Luders et al. 2010
Germany	Southern North Sea—Anglo Dutch	25	12	Grobe and Machel 2002
Hungary	Pannonian	34	129	Varsanyi et al. 1997; Varsanyi and Kovacs 2009; Rowland et al. 2011
Iran	Zagros Foldbelt	36	98	Mirnejad et al. 2011; Bagheri et al. 2014; Boschetti et al. 2020
Israel	Sinai—Levant	35	22	Chan et al. 2002
Italy	Po	33	36	Boschetti et al. 2011
Mexico	Salinas—Sureste	21	32	Birkle et al. 2002; Birkle et al. 2009
Russia	Anabar—Olenek	39	9	Alexeev et al. 2020
Russia	Nepa-Botuoba	38	7	Alexeev et al. 2020
Russia	Tunguska	37	8	Alexeev et al. 2020
Russia	Vilvuy	40	6	Alexeev et al. 2020
Spain	Betic Cordillera	23	75	Sanchez et al. 1999
Thailand	Pattani	44	20	Lundegard and Trevena 1990
USA	Appalachian	17	184	Sanders 1991; Dresel and Rose 2010; Skeen 2010
USA	Black Warrior	19	2	Collins 1976
USA	Delaware	9	70	Bodine and Jones 1990
USA	East Texas Salt	11	5	Collins 1976
USA	Forest City	14	23	Banner et al. 1989
USA	Georges Bank	18	14	Hogan and Blum 2003
USA	Green River	4	5	Collins 1976
USA	Gulf Coast Tertiary	12	330	Collins 1976; Macpherson 1989; Land and Macpherson 1992; Land 1995
USA	Illinois	15	144	Stueber and Walter 1991; Stueber et al. 1993; Demir and Seyler 1999
USA	Michigan	16	112	Wilson and Long 1993
USA	Midland—Permian	8	57	Stueber et al. 1998
USA	Mississippi Salt	13	62	Carpenter et al. 1974; Collins 1976
USA	North Louisiana Salt	10	105	Moldovanyi and Walter 1992
USA	Northeast Gulf Salt	20	2	Collins 1976
USA	Salton Trough	7	15	Thompson and Fournier 1988; Williams and McKidden 1989
USA	San Joaquin	6	66	Merino et al. 1975; Fisher and Boles 1990
USA	Williston	3	38	Collins 1976; Peterman et al. 2016
USA	Wind River	5	5	Collins 1976
Venezuela	Maracaibo	22	10	Boschetti et al. 2016

The identification number refers to Fig. 1. References are listed in the Supplementary references section.

The depth of water sampling (in metres) along the drill holes is reported as a function of the ground surface or the seafloor (for onshore and offshore basins, respectively). When available from the published sources, the sample temperature (in °C) is measured in the drill holes by wireline thermometers or, by default, estimated from the local geothermal gradient or chemical geothermometers. Besides lithium concentrations, the database also includes Mg/Li mass ratio. The salinity expressed as total dissolved solids (TDS) based on the sum of concentrations of major ions (Na^+^, K^+^, Ca^2+^, Mg^2+^, Cl^-^, SO_4_^2-^, NO_3_^−^, F^−^, HCO_3_^−^, CO_3_^2−^), was calculated from the published source data and expressed in mg l^−1^. Lithium and magnesium concentrations from about 500 published water analyses from about 44 closed basins (salars and salt lakes) from South America and China were also compiled. They are hereafter referred to as “closed basin” fluids. A posteriori quality control analysis could not be reasonably carried out on the published data used in this database.

The reservoirs on which the lithium resources were estimated combine: (1) relatively elevated median lithium concentration (> 10 mg l^−1^), (2) a relatively homogeneous lithium concentration (i.e. lower and upper quartiles in the same order of magnitude), (3) number of samples per reservoir above five and (4) known geographic location and depth of samples. The values of all parameters are available from the published sources (Table 2). A resource is “a concentration of naturally occurring solid, liquid, or gaseous material in or on the Earth’s crust in such form and amount that economic extraction of a commodity from the concentration is currently or potentially feasible”^1^. Here, our resource calculation provide the total amount of lithium contained in the considered reservoirs, without considering the storage coefficient and lithium recovery rates. The lithium resources (R_Li_ in Mtons) of the selected reservoirs were estimated using two main parameters: (1) the volume of water in the reservoir, estimated by considering the surface area (A in km^2^), the thickness (T in m) and the porosity (P, expressed between 0 and 1 where 1 corresponds to a porosity of 100 %) of the reservoirs, and (2) the water density (D, either provided in the published sources or conservatively set at 1 g.cm^-3^ by default) and the lithium concentration (C in mg l^−1^). The following equation was used^17^:

1 $${\text{R}}_{{{\text{Li}}}} = {\text{A}} \times {\text{T}} \times {\text{P}} \times {\text{D}} \times {\text{C}}$$

The surface area is the most sensitive parameter in Eq. (1). Therefore, given that storage coefficient and lithium recovery rate were not taken into account, minimizing the volume of the lithium-rich reservoirs is crucial to provide reasonably conservative resource estimates. The total surface area of the considered reservoir was not taken into account. A circle with a radius of 2 km around each sample site (i.e. drill hole) was supposed to define the area related to a single drill hole. When drill holes are less than 4 km to each other so that circles overlap, the surface area associated with the group of drill holes was deemed equivalent to that of the smallest circle enclosing the considered group of drill holes. Then, the total surface area (A in Eq. (1)) for a given reservoir was estimated according to the sum of the surface areas considered for the different drill holes and groups of drill holes. The conservative value for reservoir thickness was estimated based on the difference between the shallowest and deepest samples. The representative lithium concentration are supposed to range between the lower and upper quartiles (Q_1_ and Q_3_ respectively) of the available data for each reservoir. The porosity values are seldom reported in the published sources. The porosity generally tends to decrease with increasing depth. However, this trend is accompanied by a wide dispersion of porosity values due to changes in lithology and cementation rate. According to reference databases of reservoir porosity in sedimentary basins, the median porosity of the siliciclastic and carbonate reservoirs evolves approximatively from 24 to 10% and 18 to 6% respectively with increasing depth^18^. Here, the range of possible porosity values considered for each reservoir is between 5 and 15% (Table 2). Simultaneously to our estimation of the lithium resources, published estimates of lithium resources in known and exploited hard-rocks, salars and sedimentary formation waters were compiled^14,17,19–29^ (Table 3).

**Table 2:** Lithium resource estimates for the considered reservoirs. Q_1_ and Q_3_: quartiles 1 and 3 respectively.

Basin	Reservoir	Age of host rocks	Porosity	Density (g cm^-3^)	Lithium concentration (mg l^−1^)	Thickness (m)	Surface area (km^2^)	Resource (Mtons)	Reference
			min–max		Q_1_–Q_3_			Scenario 1–Scenario 2	
Appalachian	Oriskany	Devonian	0.05–0.15	1	148–277	2101.5	92.7	1.44–8.09	44
	Medina	Silurian		1.14	44–75	132	40.3	0.01–0.07	45
Salton Trough	Salton Sea Geothermal System	Quaternary		1.12	48–206	140	37.4	0.01–0.18	46
North Louisiana Salt	Atlanta	Jurassic		1.2	194–236	90	20.6	0.02–0.08	47
West Canadian—Alberta	Basal Quartz	Cretaceous		1.1	11–15	640	40	0.02–0.06	48
	Leduc	Devonian		1.1	20–34	651	75.4	0.05–0.28	
Rhine Graben	Landau	Paleocene		1.04	15–24	674	13	0.01–0.03	49

**Table 3:** Lithium concentrations and lithium resources compiled from published sources of hard-rock deposits, sedimentary formation waters and closed basin brines.

Type	Country	Deposits	Lithium concentration	Resource	Reference
			(Min–Max, ppm)	(Min–Max, Mtons)	
Hard rocks	Austria	Koralpe	7500–7800	0.075–0.1	20–21–22
	Australia	Greenbushes	12,200–19,000	0.255–0.857	19–21–22
	Australia	Mt Cattlin Creek	5000	0.0645–0.092	19–21–22
	Australia	Mt Marion	6500	0.0198–0.069	19–21–22
	Brazil	Volta Grande: Mibra	4600	0.1–0.9	17–22–14
	Canada	Bernic lake	6400–12,800	0.0186–0.14	19–21
	Canada	Georgia lake	4500	0.035	22
	Canada	James Bay: Cyr	5800	0.129	22
	Canada	Lacome: Quebec lithium	5300–5500	0.574	19–22
	Canada	La Corne	5200	0.1–0.3661	19–21
	Canada	La Motte	5000	0.0226–1.023	19–21–14
	Canada	Moblan	7000–17,000	0.037–0.04	22–14
	Canada	Mosse	10,400	0.003–0.016	23–22
	Canada	Separation Rapids: Big Mack	6200	0.072	22
	Canada	Separation Rapids: Big Whopper	6900	0.002–0.072	22–23–14
	Canada	Wekusko Lake	6000–7900	0.024–0.072	21–22
	Canada	Whabouchi	7100	0.21	22
	Canada	Yellowknife	6600–7900	0.1–0.129	19–14
	China	Daoxian	2600–5500	0.125–0.182	24–21–22–14
	China	Yichun	20,000	0.325–0.513	24–21–23
	China	Jiajika	5900–6000	0.204–0.48	19–21–17
	China	Lushi	4600	0.093	22
	Congo	Kitotolo	6000	0.31–0.8	24–14
	Congo	Manono	5800–6000	0.835–3.1	24–21–22
	Ireland	Blackstairs Mt: Leinster	6300	0.013	22
	Finland	Lantta: Oukovesi	4300	0.013–0.68	22–25–14
	France	Treguebbec	3300	0.13	22
	France	Echassières	3600	0.031	22
	Mali	Bougouni	14,000	0.004–0.026	19–21–22
	Namibia	Karibib	9300–14,000	0.0115–0.15	21–14
	Portugal	Brarroso–Alvao	3700–7700	0.01–0.046	19–23–22
	Russia	Alakhinskoye	5100	0.046–0.093	23–22
	Russia	Belorechenskoye	5200	0.046–0.074	23–22
	Russia	Etykinskoe	2300–7900	0.046	19–23
	Russia	Goltsovoe	3700	0.139–0.288	17–23–22
	Russia	Kolmozerskoye	5300	0.288–0.844	17–23–22
	Russia	Kosterskoye	2800	0.465	22
	Russia	Polmostundrovskoye	5800	0.1–0.363	17–23–14
	Russia	Tastygskoye	6800	0.046–0.279	23–22
	Russia	Uriskskoye	5100	0.167	22
	Russia	Vishnyakovskoe	4900	0.046–0.21	23–14
	Russia	Zavitinskoye	3200	0.05–0.46	22–23–14
	Serbia	Jadar valley	8400	0.85–1.048	21–22
	Spain	Mina Feli	5000	0.005	14
	Spain	Doade: Presqueiras	2500	0.025	22
	Sweden	Jarkvissle	4500	0.003	14
	USA	McDermitt: Kings River Valley	2400–5300	0.114–2	20–19–23
	USA	Bessemer City	6700	0.335–0.42	22–14
	USA	Kings Mountain belt	6800–6900	0.2–5.8	19–17–26
	Zimbabwe	Bikita	5800–14,000	0.023–0.17	20–22–14
	Zimbabwe	Kamativi	2800	0.28	14

	Country	Reservoir	Lithium concentration	Resource	Reference
			(Min–Max, mg l^−1^)	(Min–Max, Mtons)	
Sedimentary formation waters	Canada	Alberta basin: Fox Creek—Beaverhill	100–120	0.515–0.589	24–22–21
	France	Rhine Graben: Soultz—Cronenbourg	180–220	0.3	27–22
	Russia	Sayan—Angara—Lena: Znamenskoye	480–480	0.033	22
	USA	Gulf Salt: Smackover	146–386	0.75–1	27–24–19
	USA	Salton Though: Salton Sea—Brawley	100–400	0.316–1	27–17–14
Closed basins brines	Argentina	Salar de Cauchari	257–620	0.9–0.926	20–21–14
	Argentina	Salar des Diablillos	556–556	0.529–0.9	22–14
	Argentina	Salar del Hombre Muerto: Felix	521–620	0.8–0.9	20–27–25–14
	Argentina	Salar del Hombre Muerto: Sal de Vida	754–754	0.3–1.359	22–14
	Argentina	Salar de Olaroz	306–900	0.156–0.325	20–25–17
	Argentina	Salar de Rincon	330–400	0.5–2.8	20–25–19–21
	Bolivia	Salar de Coipasa	243–350	0.2	27–25
	Bolivia	Salar de Uyuni	187–960	5.5–10.2	24–17–28–14
	Chile	Salar de Atacama	1400–1570	3–35.7	27–25–29–19
	Chile	Salar de Maricunja	920–1240	0.2–0.44	19–21–22–14
	China	Dangxiongcuo (DXC)	330–500	0.1–0.2	19–21–14
	China	Qinghai: Taijinaier—Da Qaidam	300–300	1–3.3	19–14
	China	Zhabuye (Salt lake)	500–1000	1.3–1.53	27–25–19
	USA	Clayton Valley: Silver Peak	200–300	0.04–0.4	29–19–23–14
	USA	Great Salt Lake	40–60	0.5–0.526	27–19–21–14
	USA	Searles Lake	50–83	0.0182–0.0316	25–19–17–14

## Lithium geochemistry in sedimentary formation waters

**Figure 2:**
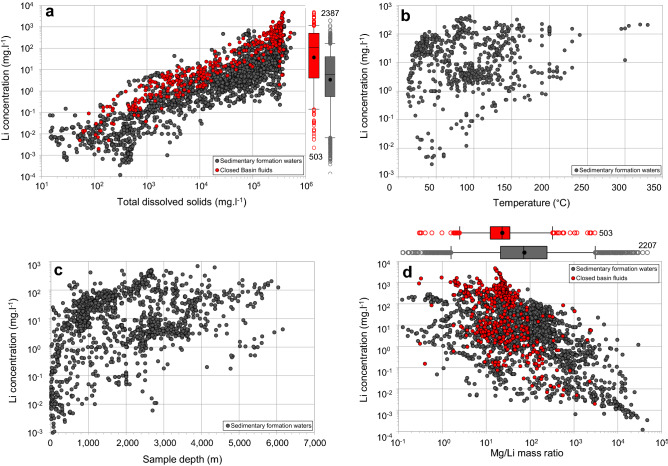
Lithium concentration in formation waters from sedimentary basins and closed basin brines worldwide as a function of various parameters. (**a**) Lithium concentration vs total dissolved solids. (**b**) Lithium concentration vs temperature. (**c**) Lithium concentration vs sample depth. (**d**) Lithium concentration versus Mg/Li mass ratio. For box plots, the lower whiskers, bottoms of boxes, central lines, circles, tops of boxes and upper whiskers represent 5th, 25th, 50th, average, 75th and 95th percentiles, respectively; circles below and above the whiskers represent outliers. The numbers refer to the number of data.

The database covers a wide range of TDS values from freshwaters (< 10^3^ mg l^−1^), to brackish waters (10^3^–10^4^ mg l^−1^), saline waters (10^4^–10^5^ mg l^−1^) and brines (> 10^5^ mg l^−1^)^30^. The ranges of lithium concentrations in sedimentary formation waters and closed basin fluids are from 1.2 × 10^–4^ to 1800 mg l^−1^ and 2 × 10^–3^ to 4720 mg l^−1^, respectively (Fig. 2). Closed basin fluids show lithium concentrations mostly between 3.8 and 488 mg l^−1^ (Q_1_ and Q_3_, respectively) while those from sedimentary formation waters classified as “brines” (i.e. TDS > 10^5^ mg l^−1^) are mostly between 0.5 and 37 mg l^−1^ (Q_1_ and Q_3_, respectively). Lithium concentrations show a positive correlation with TDS values (Fig. 2a). However, an extensive range of 2–3 orders of magnitude in lithium concentrations is observed in sedimentary formation waters for a given TDS value. The lithium concentrations in the closed basin fluids overlie the highest lithium concentrations in the sedimentary formation waters for a given TDS value. Some sedimentary formation waters have, therefore, similar lithium concentrations to those found in currently exploited salars.

In sedimentary formation waters, high lithium concentrations (> 10^2^ mg l^−1^) may be encountered from about 400 to 6000 m and 20 to 300 °C but there is no overall correlation (Fig. 2b,c). However, the lowest values of the lithium-temperature pairs (Fig. 2b) define a pseudo-trend of increasing lithium with temperature.

Most of the Mg/Li ratio in sedimentary formation waters are between 14 and 218 compared to 12 to 34 in closed basin fluids (Q_1_ and Q_3_, respectively). Lithium concentration shows a rough negative correlation with Mg/Li ratios, although the data are widely scattered (Fig. 2d). In closed basin brines, lithium extraction is more favourable when Mg/Li ratio is lower than 10 as the magnesium prevents the formation of lithium chloride, which is the first step towards forming the desired end product lithium carbonate^14,15^. For example, brines from the salar of Uyuni show an Mg/Li ratio of 19, much higher than those in the salars of Atacama (6.4) and del Hombre Muerto (1.4)^19,22^. For high-Mg brines such as those from salt lakes where Mg/Li exceeds 70, new technologies have been developed such as solvent extraction, adsorption and nanofiltration^15^.

**Figure 3:**
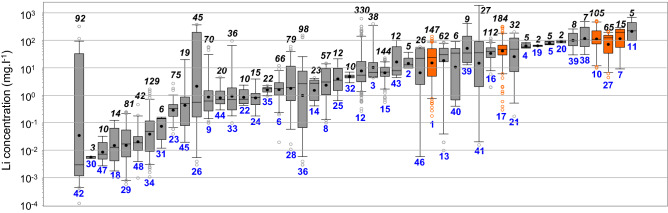
Lithium concentration in formation waters from sedimentary basins worldwide. Each boxplot represents one basin. The box plots are sorted by the increasing median value of Li concentration. The numbers in blue refer to the identification number of the basins (Fig. 1, Table 1). The numbers in black and italics refer to the number of data. Orange box plots indicate the sedimentary basins hosting the reservoirs considered for the resource estimation (see Fig. 5, Table 2). Lower whiskers, bottoms of boxes, central lines, circles, tops of boxes and upper whiskers represent 5th, 25th, 50th, average, 75th and 95th percentiles, respectively; circles below and above the whiskers represent outliers.

Median values for lithium concentration per basin range from 3.10^–3^ to 224 mg l^−1^ (Fig. 3). At the scale of a single basin, the distribution of lithium concentration may be rather homogeneous (i.e. lower and upper quartiles in the same order of magnitude) or highly scattered (i.e. over two orders of magnitude difference between the lower and upper quartiles) (Fig. 3).

**Figure 4:**
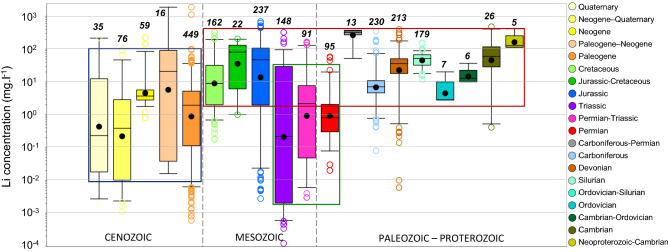
Lithium concentration in formation waters from sedimentary basins worldwide as a function of the age of the host reservoirs. The numbers in black and italics refer to the number of data. Lower whiskers, bottoms of boxes, central lines, circles, tops of boxes and upper whiskers represent 5th, 25th, 50th, average, 75th and 95th percentiles, respectively; circles below and above the whiskers represent outliers. The significances of colored squares are discussed in the lithium geochemistry in sedimentary formation waters section.

Specific ranges of lithium concentrations are distinguished according to the host-rock age of the reservoirs (Fig. 4). Similar median lithium concentrations (3 to 320 mg l^−1^) are found in waters from reservoirs located in formations from the Mesozoic and Palaeozoic—Proterozoic Era, at the exception of reservoirs in the Permo-Triassic series, where they are one to two orders of magnitude lower (0.2 to 2 mg l^−1^). They are also lower (0.23 to 21 mg l^−1^) and more scattered in reservoirs from the Cenozoic Era than all others.

It is noteworthy that most of the reported lithium concentrations are way above the typical concentration of river waters (10^–4^–0.1 mg l^−1^)^31^ and seawater (0.17 mg l^−1^)^32^, which are the most usual types of waters in the porosity at the time of sediment deposition (i.e. “connate waters”^6^). The high salinity of surface waters usually results from subaerial evaporation of seawater in sebkhras or continental waters in salars. As lithium is not significantly incorporated in evaporitic minerals, seawater evaporation enriches lithium up to 24.5 mg l^−1^^33^, thus by a factor of around 100, which cannot explain the highest values reported here. Different processes are needed to explain the high lithium concentrations, such as water–rock interactions^34–40^ along their flow path. Phyllosilicates and volcanic glass are suspected of contributing to the release of lithium, as their lithium content may be significant^34–39^. Additional sources could be clays^38,39^ and carbonates^40^. As the precise mechanisms of lithium enrichment in sedimentary formation waters remain unclear, more experimental work, aided by lithium isotope geochemistry^34,40^, could help to understand lithium release from host rocks. Besides, other processes may decrease lithium concentrations, such as diluting high-salinity sedimentary formation waters by meteoric water, diagenetic water or seawater^41^ and lithium scavenging by clay minerals^42,43^.

## Potential lithium resources in sedimentary formations waters

**Figure 5:**
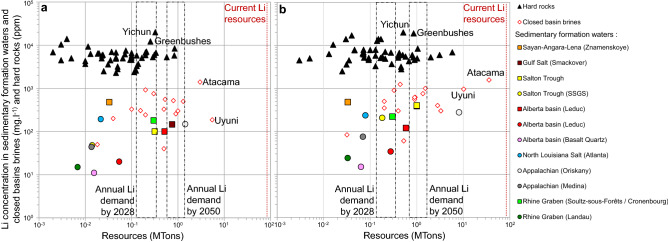
Lithium concentrations versus estimated lithium resources. The data used are reported in Tables 2 and 3 (**a**) data according to scenario 1 (this study) and lowest available resource estimates (published sources). (**b**) Data according to scenario 2 (this study) and highest available resource estimates (published sources). Hard-rock deposits (triangles); closed basin brines (diamonds); sedimentary formation waters from published sources (squares)^14,17,19–29^ and this study (circles).

Estimates of lithium resources were obtained using two scenarios (Tables 2, 3, Fig. 5). Scenario 1 (Sc1) uses lithium concentration equivalent to quartile 1 in the considered reservoir and a porosity value of 0.05, therefore minimizing the resource estimates. Scenario 2 (Sc2) uses lithium concentration equivalent to quartile 3 and a porosity value of 0.15, thus maximizing the resource estimates. In all cases, the resource estimation is conservative as it is based on reasonably minimized reservoir volume, which is the most sensitive parameter in Eq. (1). Resources estimates from Sc1 and Sc2 are shown in Fig. 5a,b respectively, with the lowest and highest available resource estimates for sedimentary formation waters, hard rocks and salars.

Seven reservoirs have been considered for the estimation of lithium resources according to the criteria and conservative approach described in the data compilation and processing section: Oriskany^44^ and Medina^45^ reservoirs in Appalachian Basin, Salton Sea Geothermal System (SSGS) in the Salton Trough^46^, Atlanta field in the North Louisiana Salt^47^, Basal quartz and Leduc formations in Alberta Basin^48^ and Landau oil well in the Rhine Graben^49^. The lithium concentrations of these seven reservoirs range between 11 and 194 mg l^−1^ and 15 and 277 mg l^−1^ (Q_1_ and Q_3_ respectively).

In Sc1, the estimated lithium resources range from 0.01 Mtons (SSGS and Landau well) to 1.44 Mtons (Oriskany reservoir). In Sc2, the estimated lithium resources range from 0.03 Mtons (Landau oil well) to 8.09 Mtons (Oriskany reservoir) (Table 2).

The estimated lithium resources of the seven considered reservoirs increase from scenario 1 to scenario 2 by a factor of 3 to 15. Although the calculation method is similar, direct comparison of the estimated lithium resources from this study with published lithium resources should be considered with caution as (1) information on input data (surface area, thickness, porosity, lithium concentration, density) is incomplete in the latter and (2) some published lithium resources include two reservoirs such as the Salton Sea and Brawley formations in the Salton Through and Leduc and Beaverhill Lake formations in the Alberta Basin. The estimated lithium resources in Sc1 and Sc2 in SSGS and Leduc formation range from 0.01 to 0.2 Mtons and from 0.05 to 0.28 Mtons. The published lithium resources range from 0.31 to 1 Mtons in Salton Trough and 0.51 to 0.59 Mtons in the Alberta Basin. In the Rhine Graben, estimated lithium resources from this study in the Landau oil well (0.01 to 0.03 Mtons according to Sc1 and Sc2) are significantly lower than published estimates for the Soultz-sous-Forêts and Cronenburg reservoirs (0.3 Mtons) (Tables 2, 3). Overall, this confirms that the approach developed in this study is conservative.

In both scenarios, the estimated and published lithium resources in the considered reservoirs are in the same range as those of hard-rock mines, and closed basin brines (ca. 10^–2^ to 10^1^ Mtons). The Oriskany reservoir (1.44 to 8.09 Mtons), Leduc formation (0.05 to 0.28 Mtons) and SSGS wells (0.01 to 0.2 Mtons) show some comparable estimated resources as some major currently exploited lithium deposits in hard rocks such as Greenbushes (0.26 to 0.86 Mtons) and Yichun (0.3 to 0.5 Mtons). In Sc2, the estimated lithium resource in the Oriskany reservoir (8.09 Mtons) is comparable to those of the most significant currently-exploited salars, namely Atacama (3 to 35.7 Mtons) and Uyuni (5.5 to 10.2 Mtons).

The forecasted global annual lithium demand ranges between 0.15 and 0.38 Mtons of lithium by 2028^2^ and between 0.6 and 1.5 Mtons of lithium by 2050^50^ (Fig. 5). The estimated lithium resources from sedimentary formation waters (Oriskany reservoir in Appalachian Basin) are in the same order of magnitude or slightly higher than the forecasted global annual demands for 2028 to 2050.

## Conclusion

Waters from some sedimentary reservoirs are characterized by high lithium concentrations similar to those from salars. Such waters are brines with salinity ten times higher than seawater. The available resources calculated using conservative hypotheses show that they are comparable to currently-exploited salars and hard-rock mines. According to our conservative estimations, the lithium resources in sedimentary formation waters of some reservoirs may exceed the global annual demand by 2028 and may reach that of 2050.

Carbon capture, utilisation and storage (CCUS), and low-enthalpy geothermal fields will enhance the production of such waters and favour the potential exploitation of such resources. The production of large quantities of non-drinkable formation waters and associated lithium recovery could finance part of the costs of the CCUS operations. Hydrometallurgical processes such as lithium separation from waters with a high Mg/Li ratio will undoubtedly benefit from further improvements.

## Supplementary Information

Supplementary Information.

## Data Availability

The published sources used for building the database are available from the corresponding author upon reasonable request.
