# Supplementary material for: Groundwater in sedimentary basins as potential lithium resource: a global prospective study
Source: Sci Rep. 2021 Oct 26;11:21091. doi: 10.1038/s41598-021-99912-7 (PMC8548349; doi:10.1038/s41598-021-99912-7)
Supplement: Supplementary file 1 — Supplementary Information. [file 41598_2021_99912_MOESM1_ESM.docx]

**Groundwater in sedimentary basins as potential lithium resource: A global prospective study**

Elza J.M. Dugamin^1*^, Antonin Richard^1^, Michel Cathelineau^1^, Marie-Christine Boiron^1^, Frank Despinois^2^, Anne Brisset^2^

1 – University of Lorraine, CNRS, CREGU, GeoRessources, Nancy, France

2 – TotalEnergies, Centre Scientifique et Technique Jean Féger, Pau, France

*Corresponding author: [elza.dugamin@univ-lorraine.fr](mailto:elza.dugamin@univ-lorraine.fr)

**Supplementary references**

Alexeev, S.V., Alexeeva, L.P. & Vakhromeev, A.G. Brines of the Siberian Platform (Russia): geochemistry and processing prospects. *Applied Geochemistry* **117,** 104588 (2020).

Andrew, A.S., Whitford, D.J., Berry, M.D., Barclay, S.A. & Giblin, A.M. Origin of salinity in produced waters from the palm valley gas field, northern territory, Australia. *Applied Geochemistry* **20,** 727‑47 (2005).

Aquilina, L. *et al*. Evolution and residence time of saline thermal fluids (Balaruc springs, Southern France): implications for fluid transfer across the continental shelf. *Chemical Geology* **192,** 1‑21 (2002).

Aquilina, L., Pauwels, H., Genter, A. & Fouillac, C. Water-rock interaction processes in the triassic sandstone and the granitic basement of the Rhine Graben: Geochemical investigation of geothermal reservoir. *Geochimica et Cosmochimica Acta* **61,** 4281-4295 (1997).

Bagheri, R. *et al.*Hydrochemical and isotopic (δ^18^O, δ ^2^H, ^87^Sr/^86^Sr, δ ^37^Cl and δ^81^Br). Evidence for the origin of saline formation water in a gas reservoir. *Chemical Geology* **384,** 62‑75 (2014).

Banner, J.L, Wasserburg, G.J, Dobson, P.F., Carpenter, A.B. & Moore, C.H.  Isotopic and trace element constraints on the origin and evolution of saline groundwaters from Central Missouri. *Geochimica et Cosmochimica Acta* **53,** 383‑98 (1989).

Birkle P., Rosillo Aragon, J.J., Portugal, E. & Fong Aguilar J.L. Evolution and origin of deep reservoir water at the Activo Luna oil field, Gulf of Mexico, Mexico. *The* *American Association of Petroleum Geologists Bulletin* **86,** 457-484 (2002).

Birkle, P., García, B.M., & Padrón, C.M.M Origin and evolution of formation water at the Jujo-Tecominoacán oil reservoir, Gulf of Mexico. Part 1: chemical evolution and water–rock interaction. *Applied Geochemistry* **24,** 543‑54 (2009).

Bodine, M. & Jones B. Normative analysis of groundwaters from the Rustler Formation associated with the Waste Isolation Pilot Plant (WIPP), Southeastern New Mexico. *The Geochemical Society Special Publication* **2,** 213-269 (1990).

Boschetti, T. *et al.* Salt waters of the Northern Apennine Foredeep Basin (Italy): origin and evolution. *Aquatic Geochemistry* **17,** 71‑108 (2011).

Boschetti, T. *et al.* Chemical and isotope composition of the oilfield brines from Mishrif Formation (southern Iraq): Diagenesis and geothermometry. *Marine and Petroleum Geology* **122,** 104637 (2020).

Boschetti, T., Angulo, B., Cabrera, F., Vásquez, J. & Montero, R.L. Hydrogeochemical characterization of oilfield waters from southeast Maracaibo Basin (Venezuela): Diagenetic effects on chemical and isotopic composition. *Marine and Petroleum Geology* **73,** 228-248 (2016).

Carpenter, A.B., Trout, M.L. & Pickett, E.E. Preliminary report on the origin and chemical evolution of lead-and zinc-rich oil field brines in Central Mississippi. *Economic Geology* **69,** 1191-1206 (1974).

Cartwright, I. *et al.* Hydrogeochimical and isotopic constraints on the origins of dryland salinity, Murray Basin, Victoria, Australia. *Applied Geochemistry* **19,** 1233-1254 (2004).

Castillo, C., Kervévan, C. & Thiéry, D. Geochemical and reactive transport modeling of the injection of cooled Triassic brines into the Dogger Aquifer (Paris Basin, France). *Geothermics* **53,** 446‑63 (2015).

Chan, L.-H., Starinsky, A. & Katz. A. The behavior of lithium and its isotopes in oilfield brines: evidence from the Heletz-Kokhav field, Israel. *Geochimica et Cosmochimica Acta* **66,** 615‑23 (2002).

Collins, G. Lithium abundances in oilfield waters in *Lithium resources and requirements by the year 2000* (ed. Vine, J.D.) Geological survey professional paper **1005**, Ch 27 (United States Government Printing Office, Washington, 1976).

Connolly, C.A., Walter, L.W., Baadsgaard, H. & Longstaffe. F.J. Origin and evolution of formation waters, Alberta Basin, Western Canada Sedimentary Basin. I. Chemistry. *Applied Geochemistry* **5,** 375‑95 (1990).

Demir, I. & Seyler, B. Chemical Composition and Geologic History of Saline Waters in Aux Vases and Cypress Formations, Illinois Basin. *Aquatic Geochemistry* **5,** 281-311 (1999).

Dresel, P.E. & Rose, A.W. Chemistry and origin of oil and gas well brines in Western Pennsylvania: Pennsylvania. *Geological Survey, 4th ser.* **48** (2010).

Eccles, D.R. & Berhane, H. Geological introduction to lithium-rich formation water with emphasis on the Fox Creek Area of West-Central Alberta (NTS 83F and 83K). *Energy Resources Conservation Board, ERCB/AGS* **10*,*** 22 (2011).

Ericken, G.E. & Salas O.R. *Geology and resources of Salars in the Central Andes* (U.S. Geological Survey, 1987).

Ericken, G.E., Chong, D.G. & Vila, G.T. Lithium resources of salars in the Central Andes in *Lithium resources and requirements by the year 2000* (ed. Vine, J.D.) Geological survey professional paper **1005**, Ch 19 (United States Government Printing Office, Washington, 1976).

Ferguson, J., Etminan, H. & Ghassemi. F. Geochemistry of deep formation waters in the Canning Basin, Western Australia, and their relationship to Zn‐Pb mineralization. *Australian Journal of Earth Sciences* **40,** 471‑483 (1993).

Fisher, J.B. & Boles. J.R. Water-rock interaction in Tertiary sandstones, San Joaquin Basin, California, U.S.A.: diagenetic controls on water composition. *Chemical Geology* **82,** 83‑101 (1990).

Fontes, J.C. & Matray, J.M. Geochemistry and Origin of Formation Brines from the Paris Basin, France 1. Brines associated with triassic salts. *Chemical Geology* **109,** 149-175 (1993).

Fontes, J.C. & Matray, J.M. Geochemistry and origin of formation brines from the Paris Basin, France 2. Saline solutions associated with oil fields. *Chemical Geology* **109,** 177-200 (1993).

Gabriela, F.M. *et al.* Chemical and isotopic features of li-rich brines from the Salar de Olaroz, Central Andes of NW Argentina. *Journal of South American Earth Sciences* **103,** 102742 (2020).

Gao, J. *et al.*Hydrochemistry of flowback water from Changning Shale Gas field and associated shallow groundwater in Southern Sichuan Basin, China: implications for the possible impact of shale gas development on groundwater quality. *Science of the Total Environment* **713**, 136594 (2020).

Grobe, M. & Machel, H.G. Saline groundwater in the Munsterland Cretaceous Basin, Germany: clues to its origin and evolution. *Marine and Petroleum Geology* **19**, 307-322 (2002).

Hitchon, B., Billings & G.K., Klovan, J.E. Geochemistry and origin of formation waters in the western Canada sedimentary basin - III. Factors controlling chemical composition. *Geochimica et Cosmochimica Acta* **35,** 567-598 (1971).

Hitchon, B., Perkins, E.H. & Gunter, W.D. Recovery of trace metals in formation waters using acid gases from natural gas. *Applied Geochemistry* **16,** 1481-1497 (2001).

Hogan, J.F. & Blum .J.D. Boron and lithium isotopes as groundwater tracers: a study at the Fresh Kills Landfill, Staten Island, New York, USA. *Applied Geochemistry* **18,** 615‑627 (2003).

Kharaka, Y.K. *et al.* Geochemistry of metal-rich brines from Central Mississippi Salt Dome Basin, U.S.A.*Applied Geochemistry* **2,** 543‑561 (1987).

Kloppmann, W. *et al.* Halite dissolution derived brines in the vicinity of a Permian Salt Dome (N German Basin). Evidence from boron, strontium, oxygen, and hydrogen isotopes. *Geochimica et Cosmochimica Acta* **65,** 4087‑4101 (2001).

Land, L.S. & Macpherson, G.L. Origin of saline formation waters, Cenozoic section, Gulf of Mexico sedimentary basin. *The* *American Association of Petroleum Geologists Bulletin* **76,** 1344-1362 (1992).

Land, L.S. Na-Ca-Cl saline formation waters, Frio Formation (Oligocene), South Texas, USA: products of diagenesis. *Geochimica et Cosmochimica Acta* **59,** 2163-2174 (1995).

Linard, Y. *et al.* Water Flow in the Oxfordian and Dogger Limestone around the Meuse/Haute-Marne Underground Research Laboratory. *Physics and Chemistry of the Earth*, Parts A/B/C **36,** 1450‑1468 (2011).

Steinmetz, R.L.L. *et al*. Northern Puna Plateau-scale survey of li brine-type deposits in the Andes of NW Argentina. *Journal of Geochemical Exploration* **190,** 26‑38 (2018).

Lüders, V. *et al*. Chemistry and isotopic composition of Rotliegend and Upper Carboniferous formation waters from the North German Basin. *Chemical Geology* **276,** 198‑208 (2010).

Lundegard, P.D. & Trevena. A.S. Sandstone diagenesis in the Pattani Basin (Gulf of Thailand): history of water-rock interaction and comparison with the Gulf of Mexico. *Applied Geochemistry* **5,** 669‑685 (1990).

Macpherson, G.J. *Lithium, boron and barium in formation waters and sediments, northwestern Gulf of Mexico sedimentary basin* (Doctoral dissertation, University of Texas at Austin, 1989).

Merino, E. Diagenesis in Tertiary sandstones from Kettleman North Dome. California -II. Interstitial solutions: distribution of aqueous species at 100°C and chemical relation to the diagenetic mineralogy. *Geochimica et Cosmochimica Acta* **39,** 1629‑1645 (1975).

Michard, G. & Bastide. J.-P. Etude géochimique de la nappe du dogger du bassin parisien. *Journal of Volcanology and Geothermal Research* **35,** 151-163 (1988).

Millot, R. & Négrel, P. Multi-Isotopic tracing (δ^7^Li, δ^11^B, ^87^Sr/^86^Sr) and chemical geothermometry: evidence from hydro-geothermal systems in France. *Chemical Geology* **244,** 664‑78 (2007).

Millot, R., Guerrot, C., Innocent, C., Négrel, Ph. & Sanjuan, B. Chemical, multi-isotopic (Li-B-Sr-U-H-O) and thermal characterization of Triassic formation waters from the Paris Basin. *Chemical geology* **283,** 226-241 (2011).

Mirnejad, H. *et al.* Major, minor element chemistry and oxygen and hydrogen isotopic compositions of Marun oil-field brines, SW Iran: Source history and economic potential. *Geological Journal* **46,** 1-9 (2011).

Moldovanyi E.P. & Walter. L.M. Regional trends in water chemistry, Smackover Formation, Southwest Arkansas: geochemical and physical controls. *The* *American Association of Petroleum Geologists Bulletin* **76,** 864-894 (1992).

Négrel, P. *et al.* Heterogeneities and interconnections in groundwaters: coupled B, Li and stable-isotope variations in a large aquifer system (Eocene sand aquifer, Southwestern France). *Chemical Geology* **296,** 83‑95 (2012).

Pauwels, H. Fouillac, C & Fouillac, A.-M. Chemistry and isotopes of deep geothermal saline fluids in the Upper Rhine Graben: Origin of compounds and water-rocks interactions. *Geochimica et Cosmochimica Acta* **57,** 2737-2749 (1993).

Peterman, Z. & Thamke, J. Chemical and isotopic changes in Williston Basin brines during long-term oil production: An example from the Poplar dome, Montana. *The* *American Association of Petroleum Geologists Bulletin* **100,** 1619-1632 (2016).

Pinti, D.L. *et al.* Fossil brines preserved in the St-Lawrence Lowlands, Québec, Canada as revealed by their chemistry and noble gas isotopes. *Geochimica et Cosmochimica Acta* **75,** 4228-4243 (2011).

von Strandmann, P.A.P. *et al.*Chemical weathering processes in the Great Artesian Basin: evidence from lithium and silicon isotopes. *Earth and Planetary Science Letters* **406,** 24‑36 (2014).

Qishun, F., Ma, H., Lai, Z., Tan, H. & Li, T. Origin and evolution of oilfield brines from Tertiary strata in Western Qaidam Basin: constraints from ^87^Sr/^86^Sr, δD, δ^18^O, δ^34^S and water chemistry. *Chinese Journal of Geochemistry* **29,** 446‑54 (2010).

Risacher, F. & Fritz, B. Quaternary geochemical evolution of the salars of Uyuni and Coipasa, Central Altiplano, Bolivia. *Chemical Geology* **90,** 211‑31 (1991).

Rowland, H.A.L. *et al.* Geochemistry and arsenic behaviour in groundwater resources of the Pannonian Basin (Hungary and Romania). *Applied Geochemistry* **26,** 1-17 (2011).

Sanders, L.L. Geochemistry of Formation Waters from the Lower Silurian Clinton Formation (Albion Sandstone), Eastern Ohio. *The* *American Association of Petroleum Geologists Bulletin* **75,** 1593-1608 (1991).

Sanjuan, B. *et al.* Major geochemical characteristics of geothermal brines from the Upper Rhine Graben granitic basement with constraints on temperature and circulation. *Chemical Geology* **428,** 27‑47 (2016).

Sanjuan, B. *et al.* Main geochemical characteristics of the deep geothermal brine at Vendenheim (Alsace, France) with constraints on temperature and fluid circulation. *Proceedings World Geothermal Congress* (2020).

Sanjuan, B., Millot, R., Dezayes, C. & Brach, M. Main characteristics of the deep geothermal brine (5km) at Soultz-sous-Forêts (France) determined using geochemical and tracer test data. *Comptes-Rendus Geoscience* **342,** 546-559 (2010).

Skeen, J.C. *Basin analysis and aqueous chemistry of fluids in the Oriskany Sandstone, Appalachian Basin, USA* (Graduate Theses, Dissertations, and Problem Reports, 2010).

Stober, I. & Bucher, K. Hydraulic and hydrochemical properties of deep sedimentary reservoirs of the Upper Rhine Graben, Europe. *Geofluids* **15,** 464-482 (2015).

Stueber, A.M. & Walter, L.M. Origin and chemical evolution of formation waters from Silurian-Devonian strata in the Illinois Basin, USA. *Geochimica et Cosmochimica Acta* **55,** 309‑325 (1991).

Stueber, A.M. Walter, L.M., Huston, T.J. & Pushkar, P. Formation waters from Mississippian-Pennsylvanian reservoirs, Illinois Basin, USA: Chemical and isotopic constraints on evolution and migration. *Geochimica et Cosmochimica Acta* **57,** 763‑84 (1993).

Stueber. A.M., Saller, A.H. & Ishida, H. Origin, migration and mixing of brines in the Permian Basin: Geochemical evidence from the Eastern central basin platform, Texas. *The* *American Association of Petroleum Geologists Bulletin* **82,** 1652-1672 (1998).

Tan, H., Rao, W., Ma, H., Chen, J. & Li. T. Hydrogen, oxygen, helium and strontium isotopic constraints on the formation of oilfield waters in the Western Qaidam Basin, China. J*ournal of Asian Earth Sciences* **40,** 651‑660 (2011).

Tellam, J.H. Hydrochemistry of the Saline Groundwaters of the Lower Mersey Basin Permo-Triassic Sandstone Aquifer, UK. *Journal of Hydrology* **165,** 45-84 (1995).

Thompson, J. M. & Fournier. R.O. Chemistry and geothermometry of brine produced From the Salton Sea scientific drill hole, Imperial Valley, California. *Journal of Geophysical Research: Solid Earth* **93,** 13165‑13173 (1988).

Varsányi, I. & Ó.Kovács, L. Origin, chemical and isotopic evolution of formation water in geopressured zones in the Pannonian Basin, Hungary. *Chemical Geology* **264,** 187‑96 (2009).

Varsanyi, I., Matray. J-M. & Kovacs, L. Geochemistry of formation waters in the Pannonian Basin (southeast Hungary). *Chemical Geology* **140,** 89-106 (1997).

Williams, A.E. & McKidden, M.A. A brine interface in the Salton Sea geothermal system, California: fluid geochemical and isotopic characteristics. *Geochimica et Cosmochimica Acta* **53,** 1905-1920 (1989).

Wilson, T.P. & Long. D.T. Geochemistry and isotope chemistry of Ca-Na-CI brines in Silurian strata, Michigan Basin, U.S.A. *Applied Geochemistry* **8,** 507-524 (1993).

Wilson, T.P. & Long. D.T. Geochemistry and isotope chemistry of Michigan Basin brines: Devonian formations. *Applied Geochemistry* **8,** 81‑100 (1993).

Xun, Z. *et al.* Evolution of the subsurface K-rich brines in the Triassic carbonates in the Sichuan Basin of China. *Groundwater* ***56,*** 832-843 (2018).

Xun, Z., Cijun, L., Xiumin, J., Qiang, D. & Lihong, T. Origin of subsurface brines in the Sichuan Basin. *Groundwater* **35,** 53-58 (1997).

Yu, X., Liu, C., Wang, C., Zhao, J. & Wang, J. Origin of geothermal waters from the Upper Cretaceous to Lower Eocene strata of the Jiangling Basin, South China: Constraints by multi-isotopic. *Applied Geochemistry* **124,** 104810 (2021).
